# A review of the flea genus *Phalacropsylla* Rothschild, 1915 (Siphonaptera, Ctenophthalmidae, Neopsyllinae, Phalacropsyllini) with new host and distributional records

**DOI:** 10.3897/zookeys.675.12347

**Published:** 2017-05-18

**Authors:** Roxana Acosta, Michael W. Hastriter

**Affiliations:** 1 Museo de Zoología “Alfonso L. Herrera”, Departamento de Biología Evolutiva, Facultad de Ciencias, Universidad Nacional Autónoma de México (UNAM), Apdo. Postal 70-399, 04510 Ciudad de Mexico, Mexico; 2 Monte L. Bean Life Science Museum, Brigham Young University, P.O. Box 20200, Provo, Utah 84602-0200, U.S.A.

**Keywords:** Host-parasite relationships, flea key, new synonymy, *Phalacropsylla* distribution

## Abstract

A redescription of the genus *Phalacropsylla* Rothschild is provided. Six species are recognized: *Phalacropsylla allos* Wagner, *P. hamata* Tipton and Mendez, *P. morlani* Eads and Campos, *P. nivalis* Barrera and Traub, *P. oregonensis* Lewis and Maser, and *P. paradisea* Rothschild. *Phalacropsylla hamata* is designated herein as a junior synonym of *P. paradisea*. The distribution of *P. paradisea* is more extensive than previously thought, extending from Arizona through southern Colorado, into New Mexico, Texas, and northern Mexico (State of Nuevo León). It is the least host-specific of all species of *Phalacropsylla*, occurring on 13 different host species including cricetid, heteromyid, murid, and sciurid rodents and several carnivores, although it most commonly occurs on *Neotoma albigula* Hartley. The range of *P. oregonensis* is expanded from eastern Oregon to southeastern Idaho. Numerous records are documented for the most common and ubiquitous species, *P. allos*, which is found in British Columbia, central to northern California, Idaho, Montana, Colorado, Nevada, Utah, Wyoming, Arizona, and New Mexico. *Neotoma cinerea* Ord is the principal host of *P. allos*. *Phalacropsylla allos* is a winter flea west of the Rocky Mountains, but it has been reported in warmer months of the year on the eastern slopes of the Rocky Mountains in Larimer County, Colorado. A distribution map and key are provided for all species in the genus *Phalacropsylla*.

## Introduction

The flea genus *Phalacropsylla* Rothschild, (Neopsyllinae: Phalacropsyllini) is represented by six species (*P. allos* Wagner, *P. hamata* Tipton and Mendez, *P. morlani* Eads and Campos, *P. nivalis* Barrera and Traub, *P. oregonensis* Lewis and Maser, and *P. paradisea* Rothschild). Recent phylogenetic studies based on morphology and molecular characters found that the tribe Phalacropsyllini may be divided in two genera: *Phalacropsylla* and *Strepsylla* Traub the former in western North America, the latter in Central America (Acosta and Morrone 2013). During the current study, many previously undocumented specimens of *Phalacropsylla* were examined from the Brigham Young University flea collection (BYUC) and the collection of the late Glenn E. Haas (now a part of the BYUC). Many of those specimens were collected from nests and those important associated host relations are discussed. The validity of several species of *Phalacropsylla* is addressed and the original generic description by Rothschild (1915) which was supplemented by Eads and Campos (1982) is expanded.

## Materials and methods

Specimens were obtained on loan from the following institutions: Brigham Young University flea collection, Monte L. Bean Life Science Museum, Brigham Young University, Provo, Utah, USA (BYUC); The Carnegie Museum of Natural History, Pittsburgh, Pennsylvania, USA (CMNH); Colección de Siphonatera, Museo de Zoología, Facultad de Ciencias, Universidad Nacional Autónoma de México, Ciudad de Mexico, Mexico (MZFC-S); the Department of Entomology, National Museum of Natural History, Smithsonian Institution, Washington, D.C., USA (USNM).

The most stable and representative characters for the genus *Phalacropsylla* are found in the modified abdominal segments of the male [T-IX (basimere and telomere), distal arm of S-IX, and the aedeagus]. The majority of *Phalacropsylla* listed under “Materials Examined” that are listed as part of the BYUC was part of the Glenn E. Haas flea collection. A designated collector for most of the fleas from his collection was not indicated on his slides but were undoubtedly collected by him. The map was prepared with ESRI^®^ ArcGIS version 10.5. Flea images were illustrated with the aid of an Olympus BX61 Compound Microscope and an Olympus CC12 digital camera accompanied with an Olympus Microsuite™ B3SV program.

## Results

### Siphonaptera

#### Ctenophthalmidae, Phalacropsyllini

##### Phalacropsylla

Taxon classificationAnimaliaSiphonapteraCtenophthalmidae

Rothschild, 1915

Phalacropsylla Rothschild, 1915: 39; Ewing, 1924: 346, 1930: 173; Jordan, 1937: 268; Ewing & Fox, 1943: 85; Hubbard, 1947: 338; Traub, 1950: 76; Hopkins & Rothschild, 1962: 299; Eads & Campos, 1982: 243–244; Holland, 1985: 125.

###### Genotype.

*Phalacropsylla paradisea* Rothschild, 1915, Paradise [Cochise County], Arizona, off *Epimys* sp. [= *Rattus*], *Mus* sp., and civet cat, collected in September, October, November, and December 1913 by Otto C. Duffner. [Note: Early collectors often referred to small sylvatic rodents as “*Mus*” and reference to the “civet cat” in southern Arizona likely refers to the ring-tailed cat (*Bassariscus astutus*, Lichtenstein) and not to skunks of the family Mephidae.]

###### Description.

Frons broadly rounded, without frontal tubercle. Inter-antennal suture (falx) well developed in both male and female. Antennal groove shallow, opened posteriorly. Antenna asymmetrical, extending onto prosternosome in male, female antenna shorter. Margin of pedicel with short setae, none extending much beyond base of clavus. Occipital area with three oblique rows of setae. Pre-antennal area (anterior to eye) with two rows of setae. Head lacking setae below or posterior to eye. Eye elliptical and pigmented; central unpigmented sinus present. Eye contiguous with two overlapping, darkly pigmented spines; lateral anterior spine broader and shorter than longer narrow mesal spine. Maxilla very elongated, extending half the length of forecoxa. Labial palpus long, extended to or beyond apex of trochanter. Pronotum with complete row of long setae anterior to 14–18 broad, bluntly pointed ctenidial spines. Mesonotal collar with several pseudosetae per side. Pleural arch well developed [an unusual characteristic for a true nest flea, Eads and Maupin (1982), Lewis and Maser (1978), and Barrera and Traub (1967)]. Suture dorsad to lateral mesonotal area expanded into a distinct rounded incrassation at posterior margin abutting pleural arch. Meso- and metasterna protruding downward producing a lobe between coxae (especially so in metasternum). Meso- and metacoxae with three long stout setae at apico-caudal margin. Fore tibia with six dorsal notches; mid- and hind tibiae each with seven dorsal notches. Distotarsomeres each with four pairs of plantar bristles with a fifth pair shifted onto plantar surface between first proximal lateral pair. More anterior terga with small marginal pigmented spinelets. Terga with two rows of slender setae; anterior row small and posterior main row long with intercalaries. Abdominal spiracles blunt at apex. Three long antesensilial bristles; middle bristle longest of three. Sensilium slightly convex; with 12 sensilial pits per side. Eighth tergum of female with caudal lobe bearing marginal row of five to seven stout long setae and six to eight short stout, more anterior setae. Eighth tergum reduced in male. Caudal margin of female S-VII with rounded dorsal lobe subtended by broad sinus. Bulga of spermatheca pyriform with slender hilla much longer than bulga. Duct of bursa copulatrix narrow, longer than spermatheca, sclerotized, with apical hyaline bursa copulatrix. Male distal arm of S-IX club-shaped; broader at apex than proximal portion. Always bearing various arrangements of spiniform setae near apex. Male basimere very broad and robust; manubrium narrow and elongated with parallel sides. Basimere not divided into lobes but may or may not possess a sinus on ventral margin. Telomere narrow (at least five times as long as widest dimension); fovea variously placed on dorsal margin. Aedeagus structurally narrower at apex than at middle. Crochet and dorsal armature on sclerotized inner tube present.

##### Phalacropsylla allos

Taxon classificationAnimaliaSiphonapteraCtenophthalmidae

Wagner, 1936

Phalacropsylla allos Wagner, 1936: 657.
Phalacropsylla monticola Augustson, 1941a: 144–145, 1941b: 156.
Phalacropsylls allos Ewing & Fox, 1943: 85; Jellison & Good, 1942: 124, 161; Jellison et al., 1943: 6, 17.
Phalacropsylla monticola Hubbard, 1943: 6.
Phalacropsylla allos Stanford, 1944: 176; Costa Lima & Hathaway, 1946: 184.
Phalacropsylla monticola Costa Lima & Hathaway, 1946: 184; Hubbard, 1947: 339.
Phalacropsylla allos Hubbard, 1947: 340–341; Holland, 1949: 9; Tipton, 1950: 65; Williams & Hoff, 1951: 313; Jellison et al., 1953: 613.
Phalacropsylla monticola Jellison et al., 1953: 613.
Phalacropsylla allos Stark, 1958: 82; Wiseman, 1955: 25; Smit & Wright, 1965: 10; Beck, 1966: 77; Hopkins & Rothschild, 1966: 301; Senger, 1966: 106; Allred, 1968: 77; Douglas, 1969: 493; Stark & Kinney, 1969: 290–293; Tipton & Saunders, 1971: 18; Weindner, 1972: 75; Haas et al., 1973: 284–285; Jellison & Senger, 1973: 67; Lewis, 1974: 153; Nelson and Smith, 1980: 274; Eads and Campos, 1982: 243–245; Campos et al., 1985: 266, 269; Holland, 1985: 125–126; Thomas, 1988: 89; Lewis et al., 1988: 91; Baird & Saunders, 1992: 9; Fagerlund et al., 2001: 95; Ford et al., 2004: 24–25, 30–31; Acosta & Morrone, 2013: 335–336, 338, 340–342.

###### Diagnosis.

**Figures 2–5.**
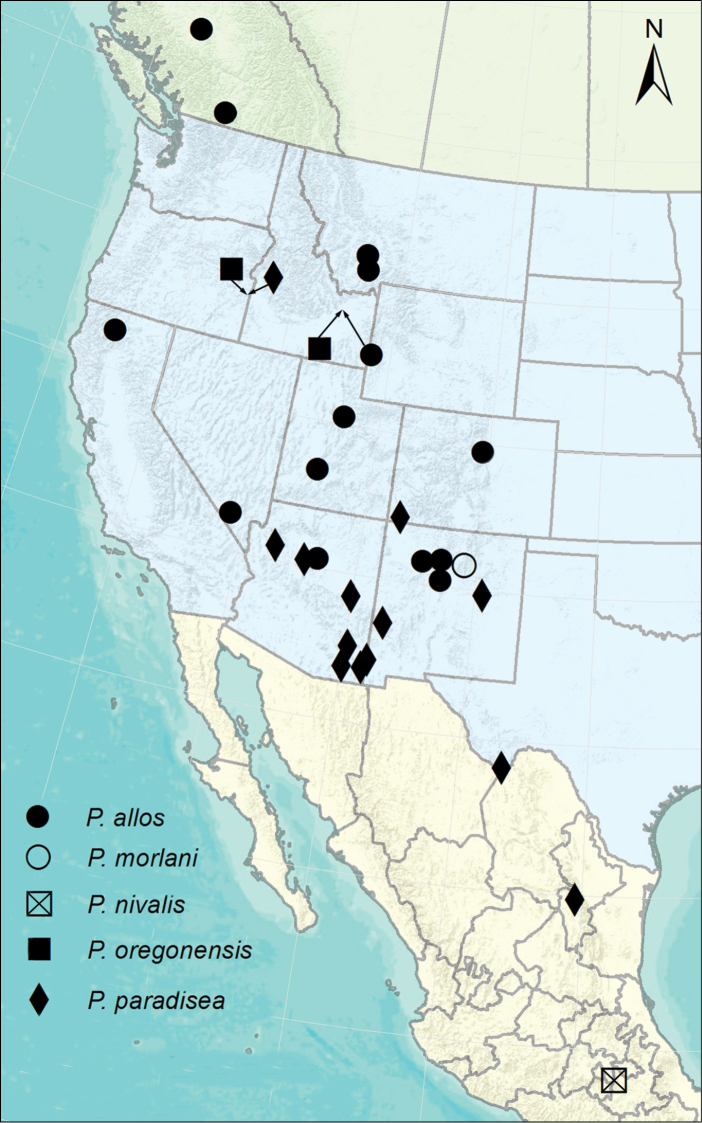
**2** *Phalacropsylla allos* male basimere and telomere. Arrows define ventral margin of basimere without sinus **3** *Phalacropsylla nivalis* male basimere, telomere, distal arm of S-IX, and aedeagus. White arrows define margin of sinus in ventral margin of basimere. AA = angular apex of basimere; HL = hyaline lobe of distal arm of S-IX **4** *Phalacropsylla oregonensis*, male holotype basimere, telomere, and distal arm of S-IX. Arrow indicates small sinus in ventral margin of basimere **5** *Phalacropsylla paradisea*, male basimere, telomere, and distal arm of S-IX. Arrows define margin of deep sinus on ventral margin of basimere. Scale = 0.2 mm

*Phalocropsylla allos* males lack a well-defined sinus in the apico-ventral margin of the basimere above acetabulum (Fig. 2). This feature is shared only with *P. oregonensis* (Fig. 4) and *P. morlani*, but may be distinguished from the former by the absence of long bent spiniform setae on the apical margin of the distal arm of S-IX and from *P. morlani* by the presence of a hyaline lobe on anterior margin of distal arm of S-IX (Fig. 3) and an acutely pointed crochet. The lobe on the caudal margin of S-VII of the females of *P. allos* and *P. nivalis* is each longer than broad, whereas the lobes of other species are broader than long. The ratio of *P. allos* is 1.9 times as long as broad, whereas *P. nivalis* is only 1.5 times as long as broad.

###### Material examined.

**USA: Arizona**, San Francisco Mts., 18 X 1989, G.E. Haas, 4♂, 4♀ (BYUC). **California**, Siskiyou County, *N. cinerea* (Ord) nest, 9 XII 1976, B.C. Nelson, 4♂, 2♀; *N. cinerea*, 31 I 1980, C.R. Smith, 1♂ (USNM). **Idaho**, Idaho Falls, National Reactor Testing Site (NRTS), Bonneville County, *N. cinerea*, 20 VIII 1967, D.E. Beck (1♂, 1♀); same data except rodent nest, 17 II 1967, (1♂); *Onychomys leucogaster* (Wied-Neuwied), 22 IX 1966, 2♀ (BYUC). **Montana**, Jefferson County, Morrison cave near White Hall, *Neotoma* sp., 31 XII 1940, H.B. Mills, 1 ♂; Ennis, in cave, *Neotoma* nest, II 1941, W.L. Jellison and G.M. Kohls, 1 ♂, 2♀ (CMNH); 1♂, 1♀ (BYUC). **Nevada**, Clark County, Spring Mts., *N. cinerea* nest, 5 VI 1985, 2♂, 2♀ (BYUC). **New Mexico**, Sandoval County, W edge Valle Grande, 2637 m, Jemez Mts., *N. cinerea* nest, 18 IX 1970, Animal Ecology Research Unit (AERU), 20♂, 16♀; Cibola County, N side Ice Cave Canyon, 2424 m, Jemez Mts., *N. cinerea* nest, 21 IX 1970, AERU, 2♂, 1♀; Sierra County, SW side Cerro del Medio, 2652 m, Jemez Mts., *N. cinerea* nest, 21 IX 1970, 1♂, 2♀; W edge Valle Grande, 2622 m, Jemez Mts., *N. cinerea* ♂, 25 IX 1970, 1♂; SE corner Baca location no. 1, Line 17, rocks with Dome Meadow, *Neotoma mexicana* Baird ♀, 16 X 1970, 1♂; Lincoln County, midden mix, 6 VIII 1991, G.E. Haas, 2♂, 1♀ (BYUC). **Utah**, Beaver County, Delano Ranger Station, *Peromyscus maniculatus* (Wagner), 25 VI 1957, D.M. Allred, 1♂, 1♀; Utah County, Provo, woodrat nest, 13 XI 1948, N.C. Acraia, 11♂, 13♀, (BYUC); same data except 2♂, 3♀ (CMNH), *N. cinerea*, 12 XI 1949, Allan Dotty, 1♂, 1♀ (USNM); Provo, Rock Canyon, *N. cinerea* nest, 15 IX 1949, V.J. Tipton, 3♂, 1♀; Provo, woodrat nest, 13 XI 1948, N.C. Acraia, 1♂, 2♀, (CMNH); *N. cinerea* nest, 12 XI 1949, A. Doty, 14♂, 21♀; Provo, Rock Canyon, *N. cinerea* nest, 13 XI 1948, 1♀; Provo, woodrat nest, 6 XI 1948, N.C. Acraia, 1♀; Provo, Rock, Canyon, *N. cinerea* nest, 24 XI 1949, D.M. Allred, 4♀; Provo, Rock Canyon, *N. cinerea* nest, 24 II 1951, D.E. Beck and D.M. Allred, 3♂; Provo, Buckley’s Mine, *N. cinerea* nest, 21 X 1950, D.M. Allred, 1♀; East of Provo, *N. cinerea* nest, 30 III 1951, D.E. Beck and D.M. Allred, 4♂, 3♀; Provo, *N. cinerea*, 25 XI 1948, N.C. Acraia, 2♂, 3♀ (BYUC).

###### Remarks.

**Figure 1.**
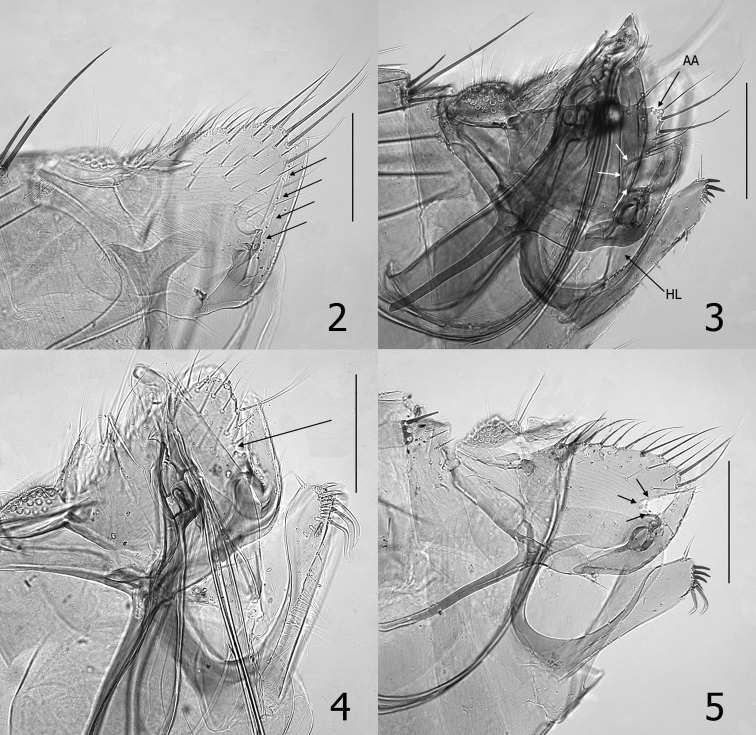
Distribution of *Phalacropsylla* species in the Canada, Mexico, and the Western United States. Arrows define same locality for two species.

*Phalacropsylla allos* is the most widely spread species of *Phalacropsylla*, occurring in southern British Columbia, Arizona, central to northern California, Colorado, Idaho, Montana, Nevada, New Mexico, Utah, and Wyoming (Fig. 1). It is sympatric with *P. paradisea* in Arizona, Colorado, and New Mexico. The vast majority of specimens examined were recorded from *N. cinerea* throughout it range, with single records from *N. mexicana* (1♂), *Onychomys leucogaster* (2♀), and *P. maniculatus* (1♂, 1♀). No other species of *Phalacropsylla* have been collected from *N. cinerea* (Table 1). Most of the specimens from nests of *N. cinerea* were collected during the cooler fall and winter months from September through February. Only one collection of three males was reported in a warmer period (June) and this site was inside a cool mine shaft (Buckley Mine) located at an elevation of 2896 m. We did not examine specimens of *P. allos* reported by Eads and Campos (1982) from *N. mexicana* (1♂), *Peromyscus difficilis* (J.A. Allen) (3♂, 1♀), *P. maniculatus* (1♂, 1♀), *Reithrodontomys megalotis* (Baird) (1♀) from Larimer County, Colorado; however, these specimens reported from the eastern slopes as *P. allos* by Eads and Campos (1982) were collected from March through August during the warmer months at elevations from 1600–1900 m. Although they collected during all months of the year, *P. allos* specimens were not collected from September through February. This seasonal disparity is enigmatic and warrants future collecting and studies.

**Table 1.** 

MAMMALIA							
ORDER	FAMILY	SPECIES	*Phalacropsylla*				
			*allos*	*morlani*	*nivalis*	*oregonensis*	*paradisea*
Carnivora	Procyonidae	Bassariscus astutus*					†X*
				
Lagomorpha	Ochotonidae	*Ochotona princeps*		†			
				
Rodentia	Cricetidae	*Neotoma albigula*					X
				
		*Neotoma cinerea*	X				
				
		*Neotoma lepida*				X	
				
		*Neotoma mexicana*	†X		X		X
				
		*Neotoma stephensi*					X
				
		*Onychomys leucogaster*	X				
				
		*Peromyscus boylii*					†
				
		*Peromyscus crinitus*				†	
				
		*Peromyscus difficilis*	†				
				
		*Peromyscus leucopus*					†
				
		*Peromyscus maniculatus*	†X			X	
				
		*Peromyscus melanotis*			X		
				
		*Peromyscus pectoralis*					X
				
		*Peromyscus truei*	†				X
				
		*Reithrodontomys megalotis*	†				
				
	Heteromyidae	*Dipodomys merriami*					X
				
	Sciuridae	*Tamiasciurus hudsonicus*					X

* Reference to “civet cat” in text likely refers to *Bassariscus astutus* and not to a skunk.

X = Represents host/parasite records for which fleas were examined in the present in study.

† = Represents host/parasite flea records reported in other studies and were nott examined in our study.

##### Phalacropsylla morlani

Taxon classificationAnimaliaSiphonapteraCtenophthalmidae

Eads & Campos, 1982

Phalacropsylla morlani Eads & Campos, 1982: 241–243; Lewis & Lewis, 1985: 149; Adams & Lewis, 1995: 68; Fagerlund et al., 2001: 95; Ford et al., 2004: 16.

###### Diagnosis.

The apico-ventral margin of the basimere is entire, without a sinus, a feature shared only by *P. allos* and *P. oregonensis* (Figs 2, 4). Readily distinguished from both by the shape of the distal arm of S-IX which is wider basally than at apex. The apex of the distal arm of S-IX (DA9) also lacks modified long spiniform setae, as in *P. allos*, whereas *P. oregonensis* possesses modified setae (Fig. 4). The female of *P. morlani* has not been described.

###### Type material examined.

**New Mexico**: Santa Fe [Santa Fe County, elev. 3048 m], *Ochotona princeps* (Richardson), 10 X 1958 [10 XI 1958 was recorded by Eads and Campos (1982:241)], H.B. Morlan, holotype (USNM).

###### Remarks.

The description of *P. morlani* was based on one male from Santa Fe County, although Eads and Campos (1982) indicated that a second male had been lost. The discrepancy in the month the holotype was collected (October or November) was not resolvable. Eads and Campos (1982) stated correctly in their diagnosis that *P. morlani* has a pronotal comb of 18 teeth but erroneously recorded 20 in their key (couplet 6.). Although *P. allos* is documented from adjacent Bernalillo and Sandoval counties, *P. allos* has not been found in Santa Fe County. Dedicated collection efforts are needed to determine if *P. allos* and *P. morlani* are sympatric at high elevations in Santa Fe County and to collect and describe the female and additional males of *P. morlani*. Since *P. morlani* was collected from a pika (*O. princeps*), additional collecting might best be directed at collecting both *O. princeps* and *N. cinerea* in Santa Fe County.

##### Phalacropsylla nivalis

Taxon classificationAnimaliaSiphonapteraCtenophthalmidae

Barrera & Traub, 1967

Phalacropsylla nivalis Barrera & Traub, 1967: 35–45; Barrera, 1968: 70, 77; Lewis, 1974: 153; Muñiz et al., 1981: 163; Ponce-Ulloa & Llorente-Bousquets, 1996: 558; Ayala-Barajas et al., 1988: 46; Adams & Lewis, 1995: 68.

###### Diagnosis.

Males of *P. nivalis* are separable from other species of *Phalacropsylla* except *P. paradisea* by the lack of a sinus in the apico-ventral margin of the basimere above the acetabulum (Figs 3, 5). Males are further distinguished from *P. paradisea* by the absence of long curved modified spiniform setae on the apex of DA9 (Figs 3, 5). See diagnosis of *P. allos* to differentiate females.

###### Material examined.

**Mexico**, **State of Mexico**, [Mirador del Poeta, N slope Mt.] Popocatépetl, 300 m SW Tlamacas, [~19.02°N, 98.38°W] 3900 m, s/*Neotoma* [*Neotoma mexicana torquata*], 19 IX 1963, A. Barrera, holotype ♂, allotype ♀ (USNM). Río Frío, 3100 m, *N. mexicana*, 17 I 1965, T. Álvarez and A. Barrera, paratype ♂; Popocatépetl, Mirador del Poeta, 3900 m, *Peromyscus melanotis* J.A. Allen and Chapman, 5 VII 1964, A. Barrera and T. Álvarez, paratype ♀, (MZFC-S).

###### Remarks.

Known only from type material from Popocatépetl mountain, Mexico. Specimens were taken from two different hosts: *Neotoma* and *Peromyscus*. *Phalacropsylla nivalis* is the most extreme southern species of the genus, occurring many hundreds of kilometers from its closest allied species, *P. paradisea*.

##### Phalacropsylla oregonensis

Taxon classificationAnimaliaSiphonapteraCtenophthalmidae

Lewis & Maser, 1978

Phalacropsylla paradisea Allred, 1968: 71 (specimen in BYUC, misidentification); Baird & Saunders, 1992: 9 (quoted misidentification of Allred, 1968).
Phalacropsylla oregonensis Lewis & Maser, 1978: 147–150; Lewis & Lewis, 1985: 149; Lewis, et al., 1988: 90; Adams & Lewis, 1995: 68.

###### Diagnosis.

Males differ from *P. paradisea* and *P. nivalis* by the absence of a sinus on the apico-ventral margin of the basimere. A small sinus is indicated, but its depth is much less than its width (Fig. 4). Readily differs from *P. allos* and *P. morlani* by the lack of modified long spiniform setae at the apex of DA9 (See diagnosis for *P. allos*). Of the two species whose lobes on the margin of S-VII are wider than long, the lobe of *P. oregonensis* is more triangular, bluntly pointed, and curved downward than that of *P. paradisea*. The latter is broadly rounded at apex.

###### Material examined.

**USA: Idaho**, Bonneville County, NRTC, Idaho Falls, *P. maniculatus*, 21 X 1966, [D.E. Beck], coll. code: 36HF, 1♂ (BYUC). **Oregon**, Malheur County, Succor Creek State Park [~43.28°N, 117.08°W], *Neotoma lepida* Thomas, 15 V 1975, C.O. Maser, holotype ♂ USNM No. 75247; same data except *P. maniculatus*, allotype ♀ [MWH re-mounted holotype and allotype, as original medium was crystalized] (USNM).

###### Remarks.

Allred (1968) recorded one male from *P. maniculatus* collected at the National Reactor Testing Station in southern Idaho as *P. paradisea* but was misidentified and is herein referred to *P. oregonensis*. This specimen and the type series from eastern Oregon are the only known representatives of this species. In addition to the specimens examined, *P. oregonensis* was also collected from *Peromyscus crinitus* (Merriam) (Lewis and Maser 1978) (Table 1). The medium in which the holotype and allotype specimens was originally mounted was crystalized, obstructing the specimens from view. The specimens were removed from the slides with xylene, remounted in Canada balsam, and are now adequately preserved.

##### Phalacropsylla paradisea

Taxon classificationAnimaliaSiphonapteraCtenophthalmidae

Rothschild, 1915

Phalacropsylla paradisea Rothschild, 1915: 39; Ewing & Fox, 1943: 85; Costa Lima and Hathaway, 1946: 184; Hubbard, 1947: 339–340; Jellison et al., 1953: 613; Allred, 1968: 71 (misidentified, see P. oregonensis); Lewis, 1974: 153; Hopkins & Rothschild, 1966: 300; Baird & Saunders, 1992: 9 (misidentified, see P. oregonensis); Fagerlund et al., 2001: 95; Acosta & Morrone, 2013: 334.
Phalacropsylla hamata Tipton & Mendez, 1968: 184–187; Lewis, 1974: 153; Eads & Maupin, 1982: 96–99; Adams & Lewis, 1995: 68; Ponce-Ulloa & Llorente-Bousquets, 1996: 558; Fagerlund et al., 2001: 95; Ford et al., 2004: 23, 29, 47. **Syn. n.**

###### Diagnosis.

Males of *P. paradisea* and *P. nivalis* each possess a deep sinus on the ventral margin of the basimere (at least as deep as wide) that separates both from other species of *Phalacropsylla*. Further separated from *P. nivalis* by the presence of long modified spiniform setae on DA9 which are absent in *P. nivalis* (Figs 3, 5). See diagnostic features of females for *P. oregonensis* above.

###### Material Examined.

**Mexico: Nuevo León**, Cerro Potosí, 3050 m, rodent nest, 20 IV 1964, V.J. Tipton et al., *P. hamata* holotype ♂ (USNM). **USA: Arizona**, Apache County, *Neotoma mexicana*, 13 XI 1973, W. Begay, 1♂ (USNM). Cochise County, China Point, Dragoon Mts., *Neotoma stephensi* Say and Ord nest, 1 X 1993, G.E. Haas, 1♂; China Point, Dragoon Mts., 19 VI 1994, G.E. Haas, 1♂, 1♀; Chiracahua, nr Paradise, *N. albigula* nest, 23 IX 1989, G.E. Haas, 8♂, 14♀; Dragoon Mts., *N. albigula* nest, 26 IX 1989, G.E. Haas, 1♂; Paradise Cemetary, Chiracahua Mts., *N. albigula* nest, 19 X 1994, G.E. Haas, 1♂, 5♀ (BYUC); Paradise, *Mus* sp., XI 1912, O.C. Duffner, 1♀, R. Traub no. B-1330 (CMNH); Paradise, *Mus* sp., IX 1913, O.C. Duffner, 1♂ lectotype; Paradise, *Epimys* sp. = *Rattus* sp., XI 1913, O.C. Duffner, 2♀ paralectotype; Paradise, *Mus* sp., 12 III 1913, O.C. Duffner, 2♂ paralectotypes; Paradise, “civit cat”, 10 IX 1913, O.C. Duffner, 1♀ paralectotype (BMNH). Coconino County, Bixler Mt., *N. mexicana*, 23 IX 1993, G.E. Haas, 3♂, 4♀; Williams, *Neotoma* nest, 13 IX 1981, G.E. Haas, 1♂, 2♀; Ben Williams, *Neotoma* nest, 20 IX 1981, G.E. Haas, 1♀; Site W-3, NNW Williams, *N. stephensi* nest, 16 X 1989, G.E. Haas, 3♂, 1♀; SE Flagstaff, *Neotoma* nest, 19 XII 1981, G.E. Haas, 3♂, 3♀; Haulapai, host unknown, I 1986, G.E. Haas, 3♂, 5♀ (BYUC). Graham County, Pinaleno Mts., host unknown, 18 V 1990, G.E. Haas, 2♂, 2♀; Pinaleno Mts., vole nest, 20 X 1990, G.E. Haas, 11♂, 10♀; Shannon Park, Pinaleno Mts., *N. mexicana* nest, 10 XI 1989, G.E. Haas, 1♀; Stockton Pass, Pinaleno Mts., host unknown, 22 XI 1989, G.E. Haas, 1♀; data missing except leg. G.E. Haas, Pinaleno Mts., 1♀ (BYUC). Greenlee County, *Dipodomys merriami* Mearns 16 XI 1938, 1♂, 1♀ (CMNH), 1♂, 1♀ (USNM). Navajo County, north of Show Low, *N. albigula* nest, 30 IX 1989, G.E. Haas, 2♂, 1♀ (BYUC). **Colorado**, Montezuma County, Mesa Verde National Park, *Peromyscus truei* (Shufedit), 20 X 1961, C. Douglas, 3♂, same data except 25 X 1961, 1♀, 26 X 1961, 2♂, 3♀, 24 XI 1961, 1♂, *P. truei/maniculatus*, 13 X 1961, 1♀ (BYUC). **New Mexico**, Bernalillo County, *N. albigula*, 20 II 1981, Curt Montman, 1♂; same data except 4 XI 1981, 1♀ (USNM, previously identified as *P. hamata*); Catron County, Ben Lilly camp ground, Mogollon Mts., *N. mexicana* nest, 23 IX 1991, G.E. Haas, 2♂, 1♀; Snow Canyon, *N. mexicana* nest, 28 IX 1996, G.E. Haas, 15♂, 18♀; Bear Wallow, *Tamiasciurus hudsonicus* (Erxleben) nest, 1 X 1998, G.E. Haas, 1♀ (BYUC). Guadalupe County, 6.5 km S of Santa Rosa, *N. albigula*, 9 X 1951, 1♂ (USNM). Hidalgo County, Peloncillo Mts., *N. albigula* nest, 23 III 92, G.E. Haas, 1♀; Peloncillo Mts., *N. albigula* nest, 24 III 92, G.E. Haas, 1♀; the Pass, Peloncillo Mts., *N. albigula* nest, 25 III 92, G.E. Haas, 3♂, 10♀ (BYUC). **Texas**, Brewster County, Big Bend National Park, 1737 m, *Peromyscus pectoralis* Osgood ♂, 2 XI 1963, V. J. Tipton et al., 2♀ (BYUC).

###### Remarks.

Tipton and Mendez (1968) described *P. hamata* from one male from Cerro Potosí, Nuevo León, Mexico. Eads and Maupin (1982) described the female of *P. hamata* from two specimens collected from Bernalillo County, New Mexico and considered an additional four males as *P. hamata*. These were collected from *Peromyscus leucopus* (Rafinesque) and *N. albigula*. With the recent accession of the Glenn E. Haas flea collection (now part of the BYUC), many specimens of *Phalacropsylla* were available for study from the vast areas of Arizona, Colorado, New Mexico, and Texas. These specimens were identified as either *P. hamata* or *P. paradisea*. While studying this material, it was impossible to distinguish females accurately from one or the other. To date, males have been distinguished primarily by the presence of various numbers of long spiniform setae on the apico-ventral margin of the distal arm of S-IX. The diagnosis of the male of *P. hamata* provided by Tipton and Mendez (1968) included: 1) A deep sinus in the caudal margin of the immovable process of the clasper (basimere), 2) four long bristles on the apical and subapical portion of basimere, 3) two hook-like spiniform setae near the apex of the distal arm of S-IX, and 4) the bifid portion of the proximal arm of S-IX as angulate. The sinus on the caudal margin of the basimere, long bristles on the apex of basimere, the number of hook-like spiniform setae present at the apex of the distal arm of S-IX, and shape of the bifid portion of proximal arm of S-IX, each proved to be quite variable within series from the same study sites and even among specimens from the same host. Based on these comparative studies, we concluded that *P. hamata* is not a valid species and consider it to be a junior synonym of *P. paradisea*. *Phalacropsylla paradisea* is representative of the genus in the southern portion of its distribution with records ranging from central Arizona, southern Colorado, through New Mexico, Texas, and into northern Mexico. *Phalacropsylla nivalis* is the only species occurring further south than *P. paradisea*. Although *P. allos* is the most commonly collected species of *Phalacropsylla*, *P. paradisea* has been collected from a much more diverse group of rodent host species (n = 10) (Table 1).

During studies on plague in the Western United States by the U.S. Army in the mid-1970s, the junior author (MWH) identified two specimens (previously unreported) of *P. paradisea* that were collected on *N. albigula* (one specimen among 37 hosts examined) and *Peromyscus boylii* (Baird) (one from 10 hosts examined) from Fort Huachuca, Cochise County, Arizona. Although the whereabouts of these two specimens are unknown, they are documented in unpublished reports of the U.S. Army Environmental Hygiene Agency-Regional Division West, Aurora, Colorado.

In the latter years of his life, Dr. Glenn Haas concentrated his studies on the fleas in nests of small mammals, primarily the nests of *Neotoma* and arboreal *Tamiasciurus*. He placed the nests in breathable paper grocery bags, maintained humidity with moist paper towels, and meticulously hand-picked the emerging adult fleas over a period of weeks and months. Thus many of his mounted specimens were teneral and often not yet expanded from their recent pupal state. These “rearing” studies document the importance of species of *Neotoma*, particularly *N. albigula*, as the primary hosts of *P. paradisea*.

#### Key to males of *Phalacropsylla*

The key to females of *Phalacropsylla* by Eads and Maupin (1982) is adequate; however, all existing keys to males requires simplification. Therefore a key for the male sex follows.

**Table:** 

1	Ventral margin of basimere without a well-defined sinus	**2**
–	Ventral margin of basimere with a well-defined sinus dorsal to the acetabulum. Sinus distinctly as deep as wide and rounded at base of sinus (*P. oregonensis* has only small sinus that is angular at base, not rounded)	**4**
2	Apical ventral margin of distal arm of S-IX with three long bent spiniform setae	***oregonensis***
–	Apex of distal arm of S-IX without long setae	**3**
3	Apex of distal arm of S-IX broadening, wider than proximal area. Anterior margin of distal arm with hyaline lobe. Crochet acutely pointed	***allos***
–	Distal arm wider at base than apex (gradual tapering towards apex). Without hyaline lobe on anterior margin of distal arm of S-IX. Crochet blunt at apex	***morlani***
4	Apex of basimere rounded. Apical ventral margin of distal arm of S-IX with two to four long, curved spiniform setae	***paradisea***
–	Apex of basimere angular. Distal arm of S-IX without modified curved setae	***nivalis***

## Supplementary Material

XML Treatment for Phalacropsylla

XML Treatment for Phalacropsylla allos

XML Treatment for Phalacropsylla morlani

XML Treatment for Phalacropsylla nivalis

XML Treatment for Phalacropsylla oregonensis

XML Treatment for Phalacropsylla paradisea
